# Intermittent electrical stimulation of the right cervical vagus nerve in salt-sensitive hypertensive rats: effects on blood pressure, arrhythmias, and ventricular electrophysiology

**DOI:** 10.14814/phy2.12476

**Published:** 2015-08-11

**Authors:** Elizabeth M Annoni, Xueyi Xie, Steven W Lee, Imad Libbus, Bruce H KenKnight, John W Osborn, Elena G Tolkacheva

**Affiliations:** 1 Department of Biomedical Engineering, University of MinnesotaMinneapolis, MN, USA; 2 Cyberonics Inc.Houston, TX, USA; 3 Department of Integrative Biology and Physiology, University of MinnesotaMinneapolis, MN, USA

**Keywords:** Arrhythmia, hypertension, optical mapping, vagus nerve stimulation

## Abstract

Hypertension (HTN) is the single greatest risk factor for potentially fatal cardiovascular diseases. One cause of HTN is inappropriately increased sympathetic nervous system activity, suggesting that restoring the autonomic nervous balance may be an effective means of HTN treatment. Here, we studied the potential of vagus nerve stimulation (VNS) to treat chronic HTN and cardiac arrhythmias through stimulation of the right cervical vagus nerve in hypertensive rats. Dahl salt-sensitive rats (*n* = 12) were given a high salt diet to induce HTN. After 6 weeks, rats were randomized into two groups: HTN-Sham and HTN-VNS, in which VNS was provided to HTN-VNS group for 4 weeks. In vivo blood pressure and electrocardiogram activities were monitored continuously by an implantable telemetry system. After 10 weeks, rats were euthanized and their hearts were extracted for ex vivo electrophysiological studies using high-resolution optical mapping. Six weeks of high salt diet significantly increased both mean arterial pressure (MAP) and pulse pressure, demonstrating successful induction of HTN in all rats. After 4 weeks of VNS treatment, the increase in MAP and the number of arrhythmia episodes in HTN-VNS rats was significantly attenuated when compared to those observed in HTN-Sham rats. VNS treatment also induced changes in electrophysiological properties of the heart, such as reduction in action potential duration (APD) during rapid drive pacing, slope of APD restitution, spatial dispersion of APD, and increase in conduction velocity of impulse propagation. Overall, these results provide further evidence for the therapeutic efficacy of VNS in HTN and HTN-related heart diseases.

## Introduction

Hypertension (HTN) is one of the strongest risk factors for sudden cardiac death (Murray and Lopez 2002). Chronic HTN affects 30–45% of the general population in Europe (Mancia et al. 2013) and more than 65 million adults in the United States, with nearly 5 million new diagnoses each year (Fields et al. 2004). HTN-induced heart diseases are the leading causes of illness and death due to the secondary complications of high arterial blood pressure. Over time, HTN leads to left atrial dilation, left ventricular hypertrophy, and impaired ventricular relaxation (i.e., “diastolic dysfunction”), and thus, affecting ventricular systole and diastolic filling, results in an increased risk of heart failure (HF) (Lalande and Johnson 2008). In addition to adverse structural changes in the myocardium, chronically elevated blood pressure also negatively affects autonomic regulation of blood pressure (Julius and Valentini 1998), heart period dynamics (Bettermann et al. 2000), and electrophysiological properties of neural and myocardial tissues of the heart, causing progressive symptom expression and elicitation of spontaneous cardiac arrhythmias. It is well known that the presence and the complexities of arrhythmias significantly influence the morbidity, mortality, and quality of life of HTN patients (Ormaetxe et al. 1993; Almendral et al. 1995; Yildirir et al. 2002; Mancia et al. 2007).

Currently, the treatment of choice of physicians for patients suffering from HTN and HTN-induced heart diseases is a regimen of antihypertensive drugs along with the adoption of appropriate dietary changes and a regular schedule of exercise (National Guideline Clearinghouse (NGC), 2010). While the aim of these treatments has been to control the rise in arterial blood pressure, it has been reported that only approximately 30–35% of patients have been shown to benefit from these treatments (Mancia et al. 2007). Resistant hypertension is defined as a lack of response to three or more antihypertensive drugs with over a fourfold greater risk of cardiovascular events compared with nonresistant hypertensive patients (Pierdomenico et al. 2005). Epstein reported that approximately 15% of his study population had resistant hypertension (Epstein 2007). As a result, there is a clear need for novel and effective alternative treatments for HTN and HTN-induced heart diseases due to the large number of patients who remain resistant to antihypertensive drugs.

Device-based approaches have been heavily considered as an effective alternative to alleviate the development of HTN and bypass some of the aforementioned issues. Several of these therapeutic device strategies act to reduce central sympathetic drive, as it is known that one cause of HTN is the inappropriate increase in sympathetic nervous activity (Sved et al. 2003; Guyenet 2006; Osborn et al. 2007). Device- and procedure-based strategies in the treatment of hypertension target either a reduction in central sympathetic drive from peripheral chemoreceptors/mechanoreceptors (baroreceptor stimulation) or a reduction in renal sympathetic efferent signaling (renal sympathetic nerve ablation). However, these approaches overlooked the heart arrhythmias (bradycardia, atrial fibrillation, atrial, and ventricular ectopic beats) which commonly accompany chronic HTN.

Vagus nerve stimulation (VNS) has the capability of restoring autonomic nervous system regulatory function not only in the vascular system but also in the heart at its neural processing hierarchy (Armour 2007). While VNS is a clinically FDA-approved therapy for depression and epilepsy, numerous studies have shown that it can be extended to treat cardiovascular diseases via its ability to modulate the parasympathetic nervous activity (Kunze 1972; Brack et al. 2011; Lazare 2013; Premchand et al. 2014). Our laboratory and others have shown that VNS is able to alter several electrophysiological properties of the heart, eventually suppressing arrhythmias and leading to an improved prognosis of HF rats, and significantly improve long-term survival after chronic HF (Harman and Reeves 1968; Xie et al. 2014).

In this present study, we investigated for the first time the potential of VNS to be used as a novel device-based therapy for HTN and HTN-induced heart diseases. Specifically, we aimed to determine the effects of VNS on the evolution of mean arterial blood pressure (MAP) and arrhythmogenesis in vivo in a genetic model of HTN: the Dahl salt-sensitive (DS) rat. In addition, at the end of the study, we investigated the effects of VNS on heart anatomy and electrophysiological properties through ex vivo high-resolution optical mapping.

## Experimental Setup

All procedures were approved by the University of Minnesota Animal Care and Use Committee and were conducted in accordance with Institutional and NIH guidelines.

Prophylactic antibiotic (gentamicin sulfate; 10 mg/kg, i.m.) was given prior to each surgery. During the surgeries, rats were anaesthetized with isoflurane (5% for  induction, 2% or 3.5% for maintenance) in oxygen (2 L/min for induction, 1 L/min for maintenance). The rat’s body temperature was maintained at 37°C on a temperature-controlled surgery table.

### Animal model protocol

**Figure 1:**
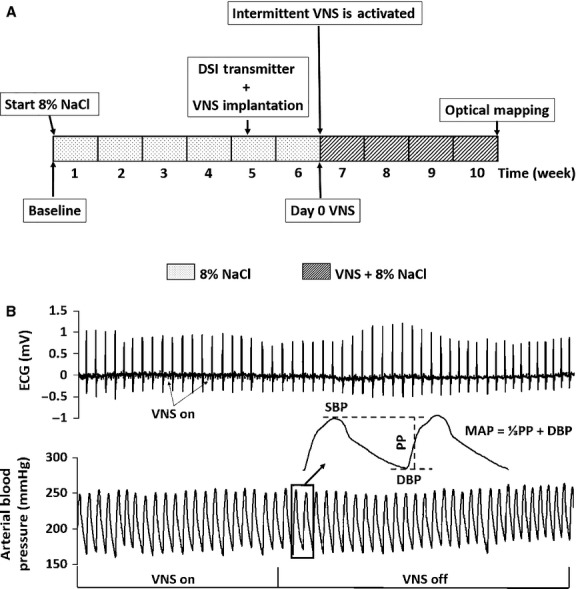
(A) Experimental design timeline. HTN was induced through 6 weeks of high salt diet (8% NaCl). DSI transmitters and VNS devices were implanted during Week 5. Rats were randomly divided into two groups: HTN-Sham (*n* = 6) with nonfunction VNS stimulators and HTN-VNS (*n* = 6) with functional stimulators. VNS therapy was turned on at Week 6, and ECG and blood pressure were monitored in both groups. (B) Sample ECG (top) and arterial blood pressure (bottom) recordings from DSI telemetric devices. Insert: MAP is calculated using SBP and DBP measured from the blood pressure recordings.

DS male rats (*n* = 12, 5 weeks old) were used for the study. HTN was induced via 8% NaCl high salt diet (S10001, Research Diets, Inc., NJ, USA) and was maintained throughout the entire duration of the study (Fig.1A). Vagus nerve stimulators (Cyberonics Model 103, Houston, TX, USA) and telemetry systems (HD-S11, Data Sciences, Inc. (DSI), Minneapolis, MN, USA) were implanted at Week 5 of the study, and rats were randomly divided into two groups: HTN-Sham (*n* = 6, with nonfunctional VNS stimulator implants of the same mass) and HTN-VNS (*n* = 6, with functional VNS stimulator implants). At the end of Week 6 (Fig.1A, “Day 0 VNS”), functional VNS stimulators were activated in the HTN-VNS group, and real-time bipolar electrograms and arterial blood pressure were recorded using DSI telemetry for both HTN-VNS and HTN-Sham groups for 4 weeks (Weeks 6 through 10).

### Vagus nerve stimulator implantation

The VNS pulse generator and lead were implanted as described previously (Shinlapawittayatorn et al. 2014; Xie et al. 2014). Briefly, the back and the neck of the rat were shaved, and the right cervical vagus nerve and common carotid artery bundle were isolated from the surrounding tissue through a small incision on the neck. Bipolar cuff electrodes were placed around the bundle. The pulse generator (8 cc, 14 g) was implanted subcutaneously and positioned on the back of the rat. Parameters of the intermittent VNS were set as follows: pulse frequency 20 Hz; pulse width 500 *μ*sec; output current 1.0 mA. The pulse generator was programmed using radio-frequency telemetry to deliver continuously cyclic VNS: 7 sec ON and 66 sec OFF. Therefore, the duty cycle was 10%. Nonfunctional stimulators were implanted in HTN-Sham group to provide the same mass (14 g).

### DSI telemetry system implantation

The DSI transmitter implantation was performed as described previously (Veitenheimer and Osborn 2011). Briefly, the surgical regions (chest and inner leg) were shaved and the transmitter pressure catheter was implanted into the descending aorta via the left femoral artery through a small incision in the inguinal area. The two ECG leads were fixed subdermally on the chest muscle. Following recovery from surgery, rats were conscious and freely moving in their home cages for the duration of the in vivo study. Continuous arterial blood pressure and ECG data acquisitions were performed with the use of a commercially available telemetry system (DSI, Inc.). The real-time bipolar electrograms and arterial blood pressure data were collected with a 500 Hz sampling rate. Each cage was placed on a receiver (model RPC1, DSI Inc.) that was connected to a computer via a Data Exchange Matrix (DSI Inc.).

### Optical mapping

Upon completion of the in vivo protocol (Fig.1A), rats were sacrificed for heart extraction through thoracotomy. The hearts were immersed in cold cardioplegic solution and Langendorff-perfused with warm (37 ± 1°C) oxygenated Tyrode’s solution as described in detail previously (Xie  et al. 2011). After 30 min of stabilization, the voltage-sensitive dye di-4-ANEPPS (5 *μ*g/mL) was added to the perfusate. Two 532 nm green lasers (Millenia Pro 5sJ, Spectra-Physics Inc., Mountain View, CA, USA) were used to illuminate both the right (RV) and left ventricles (LV) of the heart, and fluorescence signal from more than 80% of total ventricular surface was captured with two 14-bit CCD cameras (Little Joe CCD39, SciMeasure Analytical Systems, Inc., GA, USA), that ran synchronously at 1000 frames per second with 80 × 80 pixel resolution (the field of view was 12 × 12 mm). Blebbistatin (10–15 *μ*M) was added to stop heart contractions and reduce motion artifacts.

Hearts were then paced periodically at progressively decreasing basic cycle length (BCL) from 200 ms in steps of 20 ms until BCL reached 90 ms or until ventricular tachycardia (VT) or fibrillation (VF) was initiated. At each BCL, 40 stimuli were applied to reach steady state. Optical mapping movies were recorded at steady state of each pacing BCL and during ventricular arrhythmias (VF/VT) from both LV and RV epicardial surfaces of the heart. The background fluorescence was subtracted from each frame, and spatial (3 × 3 pixels) and temporal (5 pixels) convolution filters were used.

### Gross morphology and histology

Tibia length (TL)-normalized heart weight (HW) was used to quantify the VNS effects on the cardiac structure to avoid the confounder induced by the fluctuations in body weight. The left leg of the rat was severed above the knee joint, and the muscle and skin of the tibia were removed by mechanical stripping. The length of the tibia from the condyles to the tip of the medial malleolus was measured using micrometer caliper. HW normalized by TL (HW/TL) was compared between HTN-Sham and HTN-VNS rats at the end of the study. The RV and LV free-wall thickness was measured after optical mapping experiments to obtain an RV/LV wall thickness ratio to quantify hypertrophy in HTN hearts.

### Data analysis

#### In vivo DSI telemetry system data analysis

Representative examples of simultaneously recorded ECG and arterial blood pressure traces obtained from the DSI telemetry system are shown in Figure1B during VNS “ON” and “OFF” periods. Note the presence of small spikes on ECG traces (arrows) during VNS “ON” period associated with signal interference (Fig.1B, top panel).

The ECG traces were used for the estimation of arrhythmia episodes using commercial software (Ponemah Life Science Suite, DSI Inc., MN, USA). Episodes of arrhythmias were counted at “Day 0 VNS” and at the end of study (Week 10) during the same time interval (4 h at night), and were presented as the number of episodes per hour per rat. Several types of arrhythmic events were observed: premature ventricular contraction (PVC), atrial fibrillation, bradycardia, and skipping beats. PVC episodes were separated from all other arrhythmic events, as PVCs can occur in a healthy rat and pose less threat to the heart than the other types of arrhythmias.

From the arterial blood pressure traces, the following parameters were calculated using commercial software (Dataquest A.R.T., DSI Inc., MN, USA): heart rate (HR), heart rate variability (HRV), systolic blood pressure (SBP), diastolic blood pressures (DBP), pulse pressure (PP), and MAP, as indicated in Figure1B. HRV was defined as 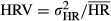, where 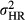 is standard deviation, and 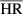 is mean HR over a 24 h time interval. ΔHR was also calculated to determine the relative changes in HR over the duration of VNS therapy. ΔHR was defined as

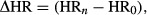


where HR_*n*_ is the HR at day *n* after VNS activation, and HR_0_ is the HR measured at “Day 0 VNS.” The relative changes in MAP were calculated as

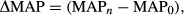


where MAP_*n*_ is the MAP at day *n* after VNS activation, and MAP_0_ is the MAP at “Day 0 VNS” (see Fig.1A). The values for ΔMAP and ΔHR were calculated separately for 12 h of day (6 AM–6 PM) and 12 h of night (6 PM–6 AM) times.

#### Ex vivo optical mapping data analysis

Optical action potential durations (APD) were measured at 80% repolarization (APD_80_), and two-dimensional (2D) APD maps were constructed to reveal the spatial distribution of APDs on both LV and RV epicardial surfaces. Mean APD was calculated at different BCLs for the visible RV and LV surfaces by averaging APDs from all pixels. The maximum slope of the APD restitution, *S*_max_, was calculated at the smallest diastolic interval. 2D maps of *S*_max_ were constructed to reveal its spatial distribution and to calculate the mean value of *S*_max_ on the epicardial surface.

The spatial dispersion of APD was estimated based on the heterogeneity index

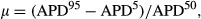
where APD^95^ and APD^5^ represent the 95th and 5th percentiles of the APD distribution, respectively, and APD^50^ is the median APD distribution.

To measure the local conduction velocity (CV), the distributions of activation times (AT) for the spatial regions of 5 × 5 pixels were fitted with the plane, and gradients of ATs *g*_*x*_ and *g*_*y*_ were calculated for each plane along the *x* and *y* axes, respectively (Xie et al. 2011). The magnitude of the local CV was calculated for each pixel as (*g*_*x*_^2^ + *g*_*y*_^2^)^−1/2^.

During sustained VT/VF, optical movies were taken every 10 sec, and the first 3000 frames (3 sec) of each episode were used for VT/VF analysis. For each VT/VF episode, fast Fourier transform was applied to each pixel to obtain the power spectrum and to determine the distribution of frequencies in the range of 5–35 Hz. The dominant frequency (DF) was defined as the frequency corresponding to the highest peak in the power spectrum (Xie et al. 2011). 2D DF maps were constructed and used to determine the maximum DF (DF_max_) on the epicardial surface, as well as the number of DF domains. The minimum size of the domain was considered to be 100 pixels with a resolution of 0.01 Hz between domains.

### Statistics

Data are presented as mean ± standard error. Statistical comparisons between HTN-Sham and HTN-VNS rats were performed using ANOVA test. *P* < 0.05 was considered to be statistically significant.

## Results

### In vivo effect of VNS on blood pressure, ECG, and arrhythmias in HTN rats

Table1 shows in vivo blood pressure and ECG 24-h mean parameters (MAP, PP, HR, and HRV) at Weeks 0, 6, 8, and 10 for all HTN-Sham and HTN-VNS rats. Table1 indicates that 6 weeks of high salt diet successfully induced HTN in both Sham and VNS groups, as indicated by significant increase in MAP and PP, as well as significant decrease in HR. Note that there were no significant differences between the groups in any parameter at either baseline or Week 6.

**Table 1:** In vivo blood pressure and ECG parameters for the all HTN-Sham and HTN-VNS rats

		Week 0 (Baseline)	Week 6: Day 0 VNS (8% NaCl)	Week 8 (8% NaCl + VNS)	Week 10 (8% NaCl + VNS)
HR (BPM)	HTN-VNS	436 ± 10	*408 ± 10	421 ± 9	*407 ± 3
	HTN-Sham	442 ± 10	*399 ± 7	*#380 ± 12	*397 ± 9
MAP (mmHg)	HTN-VNS	120 ± 3.5	*157 ± 6.0	*†171 ± 10.4	*†179 ± 11.6
	HTN-Sham	112 ± 2.5	*155 ± 11.7	*†174 ± 10.0	*†200 ± 15.7
PP (mmHg)	HTN-VNS	47 ± 2.8	*56 ± 2.7	*†62 ± 3.0	*60 ± 1.6
	HTN-Sham	38 ± 5.7	*59 ± 3.4	*58 ± 4.0	*61 ± 3.0
HRV	HTN-VNS	0.091 ± 0.009	0.090 ± 0.007	0.097 ± 0.012	0.108 ± 0.021
	HTN-Sham	0.099 ± 0.006	0.097 ± 0.009	*†0.119 ± 0.004	0.099 ± 0.015

* Denotes significance with respect to Week 0 (*P* < 0.05).

† Denotes significance with respect to Week 6 (*P* < 0.05).

# Denotes significance between HTN-Sham and HTN-VNS groups (*P* < 0.05).

**Figure 2:**
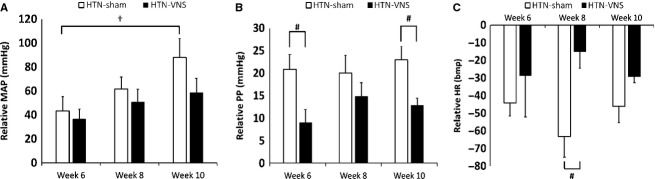
Relative change of 24-h mean data for (A) MAP; (B) PP; and (C) HR with respect to baseline data (Week 0) for both HTN-Sham and HTN-VNS rats. † indicates statistical significance (*P* < 0.05) with respect to Week 6; # indicates statistical significance (*P* < 0.05) between HTN-VNS and HTN-Sham.

Figure2 shows relative changes of 24-h mean data for MAP (panel A), PP (panel B), and HR (panel C) with respect to baseline (Week 0) for both Sham and VNS groups. The data are shown at different times during VNS stimulation: Week 6 (“Day 0 VNS”), Weeks 8 and 10 (after 2 and 4 weeks of VNS, respectively). Figure2 shows that further development of HTN (Weeks 8 and 10 in comparison to Week 6) did not affect relative change in HR and PP, but significantly increased MAP in HTN-Sham (see “†” in Fig.2A) but not in HTN-VNS group. Note that the relative changes in HR and PP are larger in HTN-Sham group in comparison to HTN-VNS group, although statistical significance is present only at some weeks (see “#” in Fig.2B and C).

**Figure 3:**
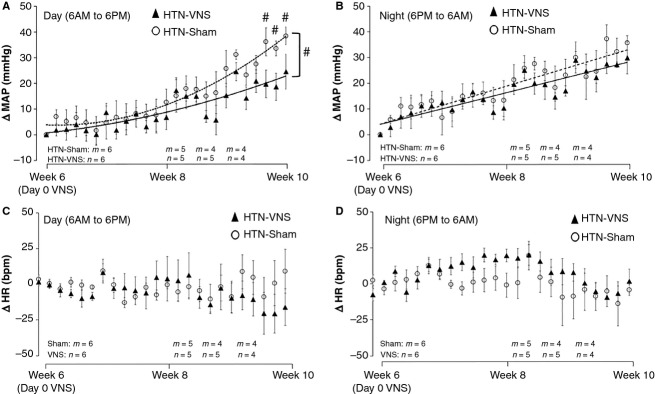
(A, B) ΔMAP and (C, D) ΔHR during the day (6 AM–6 PM) (panels A and C) and night (6 PM-6 AM) (panels B and D) for the HTN-Sham and HTN-VNS rats. # indicates statistical significance (*P* < 0.05) between HTN-VNS and HTN-Sham.

To further investigate the effect of VNS on MAP and HR, we calculated ΔMAP and ΔHR, which reflect relative change in MAP and HR at each day with respect to “Day 0 VNS.” Figure3 shows the change in ΔMAP (Panels A, B) and ΔHR (Panels C, D) for the entire duration of VNS stimulation separately for the day (6 AM–6 PM) and night (6 PM–6 AM) time periods, as rats were housed in rooms that have automatic controlled light–dark cycles. Figure3A shows that long-term VNS treatment attenuated the increase in ΔMAP during the daytime interval, where parasympathetic system is playing the dominant role in rats. This effect becomes statistically significant toward the end of the study (*P* < 0.05, see “#” in Fig.3A). On the other hand, VNS has no significant effect on ΔMAP during night period (see Fig.3B), where sympathetic activities are dominant. Figure3C,D indicate that the ΔHR in both HTN-Sham and HTN-VNS groups has similar trend over time suggesting no chronic effect of VNS on HR, which is expected since the VNS ON time (duty cycle) was only 10%.

**Figure 4:**
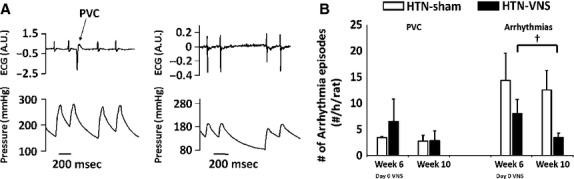
(A) Examples of episodes of PVC (left) and other arrhythmias (atrial fibrillation and skipped beats, right) from HTN-Sham rat at Week 6. (B) Mean number of episodes of PVCs and other arrhythmias for Week 6 and Week 10 for HTN-Sham and HTN-VNS rats. † indicates statistical significance (*P* < 0.05) between Week 6 and Week 10.

Lastly, we investigated the effects of VNS on the development of arrhythmias that are induced in rats as a result of HTN. Figure4A shows simultaneous recordings of arterial blood pressure and ECG from an implantable DSI transmitter for HTN-Sham rat, demonstrating examples of PVC (left) and atrial fibrillation (right). Similar types of abnormal cardiac behaviors were observed in HTN-VNS, however, with a smaller number of instances. Figure4B shows the total numbers of PVCs and arrhythmias episodes counted in both groups at Week 6 (“Day 0 VNS”) and Week 10. Note the reduction in both PVCs and other arrhythmic episodes (*P* < 0.05) in VNS rats, indicating the antiarrhythmic effects of VNS therapy in HTN rats.

### Ex vivo effect of VNS on electrophysiological properties of the HTN hearts

**Figure 5:**
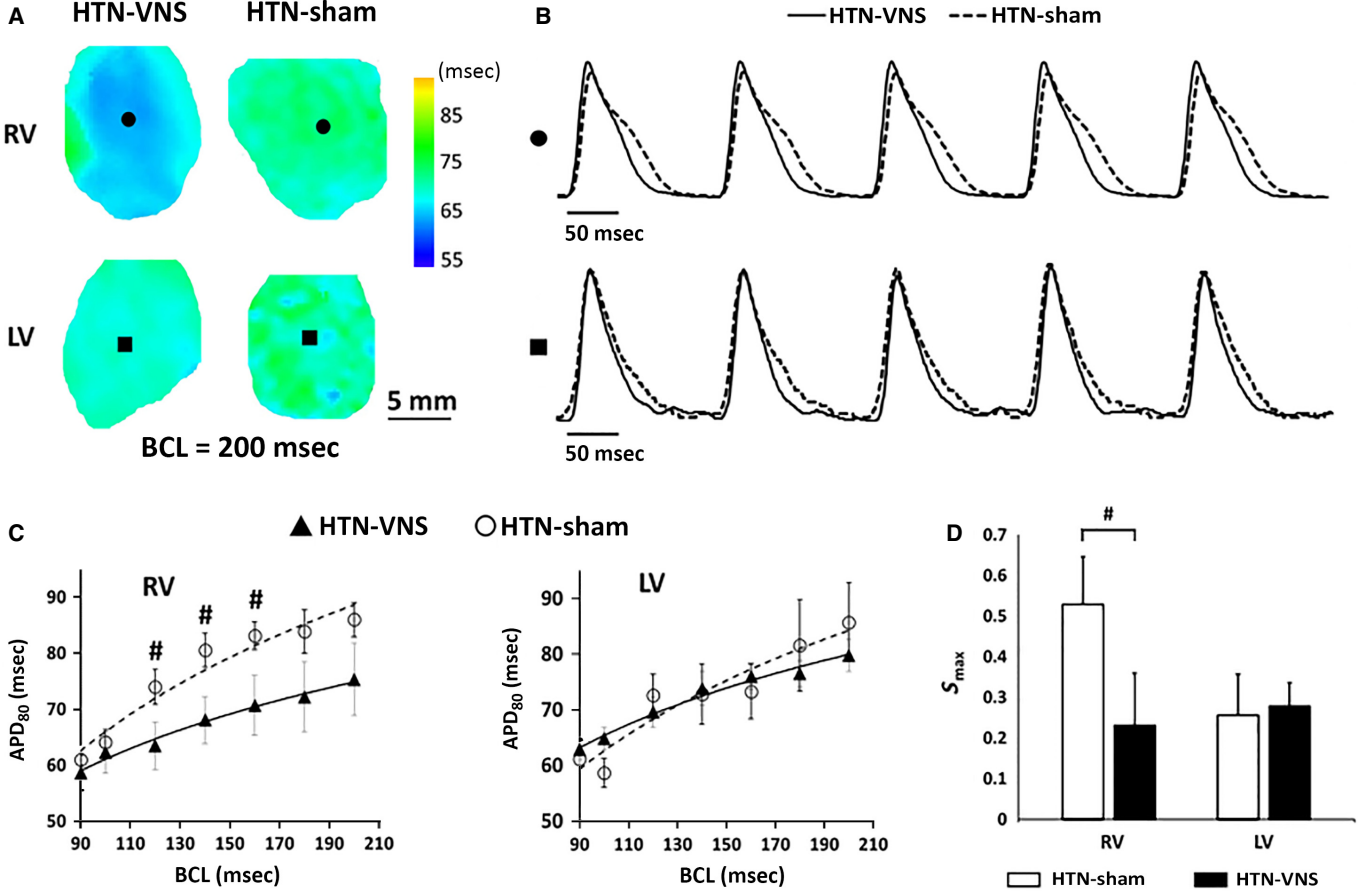
(A) Representative examples of 2D APD_80_ maps calculated at BCL = 200 ms for RV and LV of HTN-VNS and HTN-Sham hearts. (B) Single pixel action potential traces taken from RV (●) and LV (■) data from panel A for HTN-VNS (solid line) and HTN-Sham (dashed line) hearts. (C) Mean APD_80_ as a function of BCL for RV and LV of HTN-VNS (*n* = 5) and HTN-Sham (*n* = 5) hearts. (D) Mean S_max_ values for RV and LV of all HTN-VNS and HTN-Sham hearts. # indicates statistical significance (*P* < 0.05) between HTN-VNS and HTN-Sham.

Typical examples of 2D APD maps of the RV and LV of HTN-Sham and HTN-VNS rat hearts obtained at BCL = 200 ms are presented in Figure5A, with corresponding traces of action potentials from a representative single pixels shown in Figure5B. Mean APD_80_ calculated in both groups for various BCLs are presented in Figure5C for RV (left panel) and LV (right panel). Note that VNS reduced APD in the RV, with significant reduction at BCLs 120, 140, and 160 ms, but not in the LV. In addition, the maximum slope of APD restitution curve (*S*_max_) was significantly reduced in the RV but not in the LV as a result of VNS therapy, as indicated in Figure5D.

**Figure 6:**
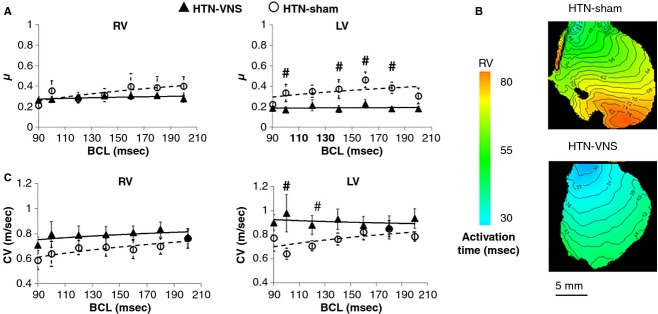
(A) APD heterogeneity index *μ* and (C) mean CV as a function of BCL for RV and LV of HTN-VNS and HTN-Sham hearts. (B) Representative examples of activation time maps calculated at BCL = 180 ms. # indicates statistical significance (*P* < 0.05) between HTN-VNS and HTN-Sham.

To determine whether VNS affected the spatial dispersion of APD, we calculated the heterogeneity index *μ* separately for RV and LV at different BCL values. Figure6A indicates that VNS significantly reduced *μ* in LV but not in RV at most BCLs.

To study whether VNS affected CV of action potential propagation, we constructed AT maps for both HTN-Sham and HTN-VNS groups at various BCLs. A representative example of AT maps at BCL = 180 ms is shown in Figure6B, indicating normal propagation in the RV of both rat hearts. Figure6C shows mean CV data at different BCLs for both groups indicating that VNS increased CV both in the RV and LV, although the effect is significant only at some BCLs.

**Figure 7:**
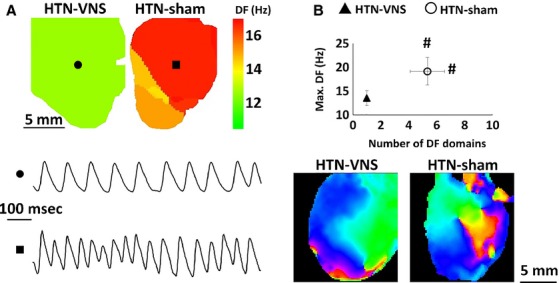
Dynamics of ex vivo arrhythmias. (A) Representative example of 2D DF map from RV of HTN-VNS and HTN-Sham hearts (top) and single pixel sample traces of VT (●) and VF (■) (bottom). (B) Maximum DF as a function of number of DF domains from all arrhythmic episodes in HTN-VNS and HTN-Sham hearts. # indicates statistical significance (*P* < 0.05) between HTN-VNS and HTN-Sham. (C) Snapshot of representative examples of phase movies during arrhythmia for HTN-VNS and HTN-Sham hearts (see supplemental material for movies).

To investigate the effect of VNS on dynamical behavior of ex vivo arrhythmias, we analyzed optical mapping movies from either spontaneous arrhythmias or the ones that were induced at the end of our pacing protocols. In all of our experiments, sustained VF was observed in all (6/6) HTN-Sham rats, while sustained VT, but not VF, was observed in 4/6 HTN-VNS rats. None (0/6) of the HTN-VNS rats developed VF, suggesting that VNS provided protection against the development of VF. The analysis of the VT/VF dynamics is shown in Figure7A–C. Figure7A shows a representative example of 2D DF maps for RV of HTN-VNS and HTN-Sham hearts (top) with the single pixel traces (bottom) clearly indicating the presence of VT/VF. Figure7B shows the DF_max_ and the number of DF domains. Note that the HTN-Sham rats not only had more DF domains but also higher DF_max_ than the HTN-VNS rats, indicating more complex dynamics of ex vivo cardiac arrhythmias. Figure7B shows representative examples of phase movies during arrhythmias in HTN-VNS and HTN-Sham hearts (see supplemental material).

Lastly, we measured the heart weight/tibia length ratio (HW/TL) and RV/LV wall thickness ratio at the end of the study. Our results show that both parameters were similar in the HTN-VNS (HW/TL: 0.38 ± 0.03; RV/LV: 0.32 ± 0.01) and HTN-Sham (HW/TL: 0.42 ± 0.01; RV/LV: 0.30 ± 0.01, *P* = N/S) groups suggesting that 4 weeks of VNS did not induced structural remodeling of the HTN heart.

## Discussion

Autonomic dysregulation is a feature of chronic heart failure and is characterized by a sustained abnormal elevation in sympathetic nerve activity and by withdrawal of parasympathetic activity (Bell and McLachlan 1979; Esler and Kaye 2000; Esler et al. 2003). Autonomic imbalance, which is reflected in reduced heart rate variability and reduced baroreflex sensitivity, is associated with worsening HF and increasing risk of mortality (La Rovere et al. 1998; Nolan et al. 1998). Modulation of parasympathetic activation with electrical stimulation is a potential therapy for HF, but has received limited attention because of its inherent complex cardiovascular effects (Gray et al. 1998; Hao et al. 2004; Hoeks et al. 2007). However, recent clinical studies have demonstrated the salutary effects of parasympathetic stimulation in HF (De Ferrari et al. 2011; Premchand et al. 2014).

In the present study, for the first time, we used a HTN rat model to characterize the effects of VNS on blood pressure and arrhythmia incidence, as well as the effects of VNS on the electrophysiological properties of the heart. Our results confirm that the VNS modulates the balance of the autonomic nervous system by promoting parasympathetic and decreasing sympathetic activity.

Our major findings are as follows: (1) 4 weeks of intermittent VNS attenuated the increase in MAP in HTN-VNS rats as compared to HTN-Sham rats; (2) VNS reduced the number of in vivo arrhythmic events, as well as the spatiotemporal complexity of ex vivo arrhythmias in HTN-VNS rats as compared to HTN-Sham rats; and (3) VNS induced beneficial changes in the electrophysiological properties of the heart, including a reduction in APD during rapid drive pacing, *μ*, and S_max_, and an increase in CV. Overall, these results provide further evidence for the therapeutic efficacy of VNS in HTN and HTN-related heart diseases.

### The effect of VNS on blood pressure

Recent studies have shown that chronic electrical stimulation of the carotid sinus activates the baroreflex and reduces MAP by inhibiting central sympathetic outflow (Lohmeier et al. 2010; Sabbah 2012). This reduction on blood pressure is substantial, and is currently being used as a clinical therapy for refractory hypertension (Heusser et al. 2010; Bakris et al. 2012).

The present study shows that chronic VNS also causes an attenuation of MAP elevation during the spontaneous development of chronic HTN, likely through a similar mechanism. In our study, we accounted for the light–dark cycle of the rats. The rationale for day-night separation is that the roles of sympathetic and parasympathetic activity are different when the rats are active (during the night) and at rest (during the day). At rest, parasympathetic activity predominates over sympathetic tone (Olshansky et al. 2008). Recently, it was demonstrated that the sympathetic and parasympathetic nervous systems are involved in diurnal variation in heart rate-corrected QT intervals (QTc) in rats (Honda et al. 2013). Specifically, the enhanced parasympathetic nervous activity during the night induces a slight but significant QTc prolongation. Our results show that VNS induced a more pronounced modulation of MAP during the daytime, when the nocturnal rats are less active, and suggest that VNS is more effective when parasympathetic nerve activity predominates and the sympathetic tone is reduced, that is, when the rat is asleep. This is an indirect indication that the parasympathetic nerve activity is impaired by HTN; and that VNS lowers MAP by enhancing parasympathetic nerve activity and restoring autonomic balance.

### The antiarrhythmic effects of VNS

The antiarrhythmic effects of VNS in ischemic HF are shown in previous studies (Schwartz et al. 1984; Zuanetti et al. 1987; Vanoli et al. 1991). In this study, we demonstrate that VNS also has antiarrhythmic effects in HTN-induced heart disease. VNS therapy significantly reduced the incidence of in vivo arrhythmias (tachycardia, bradycardia, and skipped beats) in HTN rats. In addition, our optical mapping results demonstrated that the complexity of spontaneous/induced ex vivo arrhythmias is significantly reduced in HTN-VNS rats as compared to HTN-Sham, which is further evidence of the antiarrhythmic effects of VNS treatment in HTN-induced heart disease.

Antiarrhythmic effects were associated with significant VNS-induced reduction in the APD and the slope of APD restitution (in RV), as well as the reduction in the spatial dispersion of APD and increase in CV (in LV) during rapid drive pacing. The electrophysiological changes that occurred due to VNS in LV are in contrast to the ones that were observed in most HF hearts. Reduction in CV and increase in *μ* have been shown to create an arrhythmogenic substrate that is able to promote the inception and preservation of reentrant arrhythmias (Qu et al. 2000; Banville and Gray 2002). In HF, conduction slowing has been linked to a decrease in expression of the gap-junction protein Connexin-43 (Dupont et al. 2001). Likewise, elevated Connexin-43 expression is correlated with faster conduction in the myocardium (van Kempen et al. 1991). Short-term VNS therapy has been previously shown to preserve the expression of gap-junctional protein Connexin-43 over the course of minutes after cardiac injury (Ando et al. 2005). Although we did not directly measure Connexin-43 expression in the LV, our results seem to agree with this finding. All of these electrophysiological changes are likely to be the underlying mechanisms to the antiarrhythmic effects of VNS.

The exact mechanisms underlying the antiarrhythmic effects of VNS are still not well studied. One possible mechanism may be due to the activation of the vagus nitric oxide (NO) pathway, since direct stimulation of the vagus nerve releases NO through a neuronal NO synthase mechanism in the ventricle. Brack et al. previously demonstrated the important role of NO in mediating the protective effects of VNS in the rabbit hearts (Brack et al. 2007, 2009). Another possible mechanism may involve reactive oxygen species (ROS). It is known that during myocardial injury, there is an increase in ROS production and oscillation of the mitochondrial membrane potential, which plays an important role in the genesis of cardiac arrhythmias (Brown and O’Rourke 2010). Shinlapawittayatorn et al. have shown that VNS was able to stabilize cardiac mitochondrial membrane potential by protecting the depolarization of mitochondrial membrane potential (Shinlapawittayatorn et al. 2014).

We should note that in our study, we only evaluated a limited number of spontaneous arrhythmic episodes, and therefore, arrhythmias were relatively scarce even in HTN-SHAM rats. Another approach, such as stressing the heart using programmed stimulation, might have led to stronger results.

### Mechanistic insights into chronic VNS therapy

At the cervical level, the vagus nerve contains a composite of myelinated A and B fibers, as well as unmyelinated C fibers, which are the most numerous (approximately 65% to 80%) (Foley and DuBois 1937). According to Woodbury et al. (Woodbury and Woodbury 1990), activation of C-fibers requires higher stimulation amplitudes and longer pulse durations than the activation of myelinated A and B fibers. For instance, Zagon et al. (Zagon and Kemeny 2000) demonstrated that myelinated vagal fibers were activated in anesthetized rats at 100 *μ*A (at 30 Hz, and a pulse width of 0.5 ms), while increasing current up to 200 *μ*A activated nonmyelinated fibers. The stimulation parameters that have been used in our study (pulse width of 0.5 ms and current of 1.0 mA) suggest that both myelinated and unmyelinated fibers are stimulated. Similar level of stimulation will also engage the vagal motor fibers, especially the myelinated fibers, according to the study of Ben-Menatchem et al. (Ben-Menachem 2002). Future studies are needed to determine whether the therapeutic mechanism of VNS predominantly occurs through the afferent or efferent neural pathways.

Our results clearly demonstrate that VNS is capable of attenuating the high MAP and inducing antiarrhythmic effects in HTN, and provides evidence for VNS as an effective device-based therapy for HTN and HTN-induced heart disease. Our results also suggest that the therapeutic effects of VNS are associated with chronic treatment, rather than acute stimulation. Indeed, the beneficial effect of VNS on attenuating high MAP became significant after only 4 weeks of chronic VNS. Therefore, further evaluation of VNS as a clinical HTN therapy requires prolonged chronic stimulation.

In this manuscript, we reported the beneficial effects of VNS on blood pressure and arrhythmia in a HTN rat model, and we investigated the effect of VNS on several electrophysiological parameters. Nevertheless, more comprehensive evaluation is required to reveal the mechanisms by which VNS affects different in vivo and ex vivo electrophysiological and structural characteristics of rats. Our current study raised a number of questions that can help in designing these future experiments.

Our results demonstrated that VNS affected RV and LV in a slightly different ways. Indeed, VNS reduced APD and S_max_ only in RV, but increased CV and decreased APD spatial dispersion predominantly in LV. This suggests that different electrical remodeling of both ventricles takes place as a result of chronic VNS. On the other hand, our data do not suggest any anatomical remodeling of both ventricles. These results are somewhat intriguing since LV (but not RV) contractile properties are expected to be the main determinant of MAP. It is unclear whether RV/LV electrophysiological remodeling is accompanied by a remodeling of the corresponding extracellular matrix.

In addition, chronic VNS may also provide an anti-inflammatory effect through the cholinergic anti-inflammatory pathway. The neural signal that is sent from the efferent fibers in the vagus nerve can inhibit the release of cytokines, and release the neurotransmitter acetylcholine. Cytokine-producing cells contain acetylcholine receptors which transduce signals resulting in the inhibition of cytokine synthesis. By decreasing the level of cytokine synthesis, the magnitude of the inflammatory response is dampened (Tracey 2007). This effect was demonstrated by Zhang et al. by measuring the plasma C-reactive protein (CRP) levels (Zhang et al. 2009). Pacing-induced heart failure promotes systemic inflammation and was shown to have increased plasma CRP. VNS treatment significantly reduced the plasma CRP.

## Conflict of Interest

Drs. Libbus and KenKnight are employees of Cyberonics, Inc.

## Supporting Information

**Video S1.** HNT-VNS_VT.

**Video S2.** HTN-Sham_VF.
